# Processes of Refrigeration Used for Dressed Beef in Transportation, Etc.

**Published:** 1892-11

**Authors:** Williamson Bryden


### PROCESSES OF REFRIGERATION USED FOR DRESSED BEEF IN TRANSPORTATION, ETC.*

By Williamson Bryden, V. S.

A few weeks ago, Prof. Schwarzkopf, chairman of the "Food Inspection" section of this organization, requested me to give a sketch of the processes of refrigeration used for dressed beef in transportation, etc. I only regret my inability to comply with the technical exactness the subject deserves.

Within the last fifteen years a vast amount of business in the transportation of fresh meats, fowl, fish, and even fruit and vegetables, has been done, that would have been impossible had it not been for the great improvements made in refrigeration, in cold storage, and the wonderful systems that have been organized for the sale and distribution of fresh products. Formerly meats had to be *salted* or *frozen* in the *North*, or *dried* in the *South;* now, the United States, New Zealand, Canada, Australia, and the River Platte, dispose of such products in the markets of Great Britain in a condition as fresh and sound as when killed.

At first, *ice* and *common salt* was used as a refrigerant, and succeeded sufficiently well to demonstrate the possibility of carrying successfully dressed meats from Chicago to Liverpool; then, however, it was necessary to use the meat as soon as possible after the exposure incident to its removal from the ship refrigerators, where it had been kept at a temperature of from 33 to 37 degrees Fahrenheit, or thereabouts.

* Report presented at the 29th annual meeting of the United States Veterinary Medical Association.

As the business increased, being in the hands of enterprising men, who were not long satisfied to supply the ports of landing only, they, in a short time, had their goods in every inland city of Great Britain.

To accomplish this required cold storage warehouses con venient to the docks, lines of refrigerated cars for inland transportation, and conveniences for its sale in every town. While this was being gradually consummated, the attention of scientific men was being directed to the necessity for some process of refrigeration less expensive, and that would produce a lower temperature than the old *ice and salt mixture*, which had sometimes proved unreliable in the hottest months. It was a process prodigal of valuable space, so important on ships, when freights are high, from the fact that (1) the ice and salt for use on the ocean voyage, (2) the room for the boxes containing the ice and salt mixture, and (3) the alley-ways between and at the ends of the rows of beef for the circulation of the cold air, and for the men in charge to work, took up about one-third of the cubic space in the beef chambers.

An exact, technical description of the different processes of preserving fresh beef, to which this article principally applies, I cannot give, neither will I weary you with the different processes of ice-making, the machinery invented to accomplish it, or to making and applying the different freezing mixtures; the history of these would date back many years. It may be stated, however, that the machines that have so far proved the best adapted for the trade on steamships, have been those which utilize the lowering of temperature caused by sudden expansion of a compressed gas, and by those that make use of a like effect when a liquid becomes volatilized.

Steamships sailing from Boston are equipped mostly either with what is called the "Haslam," or cold blast, or with the "Kilburn," a type of the ammonia process, in which anhydrous ammonia is used, and which at 15 degrees absolute pressure has a boiling point 72 degrees below the freezing point of water. The mechanical part consists of a condenser and a refrigerator, both made of welded steel, and containing coils of iron pipes; also two pumps, one for the condensing sea water, the other for circulating the brine and two compressors. The supply of ammonia is kept in a drum; when the machine is to be charged this ammonia first enters the refrigerator, from which it passes into the compressor, then into a coil in the condenser. Here it is subjected to a deluge of cold water which is pumped into the condenser shell; this partly cools the condensed ammonia. By the action of the compressor pump the pressure on the liquid is removed and it enters the shell of the refrigerator in the form of a gas or vapor which has become intensely cold from its sudden expansion. This refrigerator contains a coil of pipes continuous with the galvanized pipes which cover the ends, sides, top and centre of the beef boxes. In these a brine made of chloride of calcium, which freezes only at a very low temperature, is kept in circulation by the brine pump until a heavy coat of ice has formed within the beef boxes. As the brine returns to the refrigerator the ammonia gas in its shell cools it or abstracts any heat the boxes may have still retained.

Several years ago I was engaged for a couple of years or so inspecting beef for shipment on ships where both the ice and salt and the Kilburn processes were used. Beef had been arriving in Liverpool in poor condition, and some misunderstanding between the manufacturers of the machines, the beef company and the steamship company was the result.

The machines had been tested in Spring, when the sea water was from 40 to 50 degrees Fahrenheit, and worked very well ; but in midsummer, when the water in the harbor and in the Gulf Stream reached over 70 degrees, the beef boxes proved to be either too large or the machines too small or weak. My inspection was expected to reveal whether any neglect or mismanagement of the beef after slaughtering or on the land journey to the ships had occurred which might, in part at least, be responsible for the quality and condition of the beef at its destination.

My duties were, therefore, to discover, if possible, (a) if the cattle were healthy and in condition to be slaughtered; (b) the carcasses properly cooled and the animal heat all removed before shipment; (c) the cars in such condition and temperature as proved that they had been fit to load, properly recharged with ice and salt at the charging stations along the route, and that they had not been sidetracked or delayed under a burning sun on the journey to port.

Ice and salt is used on the cars, and there is a box at each end of the car, which is charged through the roof ; each car carries about one hundred quarters of beef, which is hung from hooks, half fore and half hind quarters, a few boxes of tongues being sometimes added. The loaded cars arrive one or two days before the ships sail, and the beef is transferred to the beef boxes on the ships as expeditiously as possible and before the ice formed on the brine pipes begins to drip or melt.

The machinery being idle on the return voyage, it becomes necessary to start it up a sufficient length of time before receiving the beef to reduce the temperature of the beef boxes on the ship so low that the ice formed on the brine pipes will not melt while the process of loading is going on. This depends on the size of the machine, the size of the beef boxes and the temperature of the sea water used in the ammonia condenser.

Before proceeding to open the cars the following temperatures had to be taken: (1) Temperature of the beef boxes on the ship before opening; (2) air; (3) sea water, ingoing and returning; (4) brine, ingoing and returning. The same are taken when the boxes are closed, and they cannot be opened until their arrival at Liverpool. The boxes hold from 1,000 to 2,000 quarters. Some of the ships are equipped with more than one box and carry over 3,000 quarters. I may here add that the refrigeration of mutton and lamb is different, from the fact that it can be frozen, while beef is best kept just above freezing. Lines of sailing ships are engaged carrying mutton, etc., from Australia, New Zealand and the River Platte, some of their cargoes reaching as high as 80,000 carcasses.

On proceeding to examine the beef in the cars, I armed myself with half a dozen or more beef thermometers, about three times as large as our clinicals, very strong, and with a scale of from 20 to 70 degrees Fahrenheit, a skewer, and as many more ordinary weather thermometers. The beef thermometers were allowed at least five minutes and the others at least fifteen minutes. On the car door being opened I entered, the door was closed on me, the thermometers inserted in the beef, and the others placed, some within eighteen inches of the top and others the same distance from the floor. The beef was also handled to detect soft spots or smell, and the sides of the car for moisture, etc. A record of all these was kept and reported to Liverpool.

On land some refrigerators of a somewhat similar principle have had artesian wells bored to obtain water at a temperature of about fifty degrees Fahrenheit for the hot months.

I regret being unable to give you a more exact and technical description of this interesting subject. It will serve, however, to illustrate one of the many duties the veterinarian may be called upon to perform outside of his regular profession.

